# The Community Faces Model: Community, University and Health Department Partners Thriving Together for Effective Health Education

**DOI:** 10.33596/coll.29

**Published:** 2019-02-22

**Authors:** Dina Drits-Esser, Heather Coulter, Meghan C. Mannello, Grant Sunada, Stephen C. Alder, Pastor France A. Davis, Doriena Lee, Valentine Mukundente, Ed Napia, Brenda Ralls, Sylvia Rickard, Fahina Tavaké-Pasi, Louisa A. Stark

**Affiliations:** 1 University of Utah, US; 2 Oregon Health and Science University, US; 3 Calvary Baptist Church, US; 4 Best of Africa, US; 5 Urban Indian Center of Salt Lake, US; 6 Utah Department of Health, US; 7 Hispanic Health Care Task Force, US; 8 National Tongan American Society, US

**Keywords:** Community health partnerships, diverse partnerships, multiple ethnic communities, model, community-based participatory research, engaged scholarship, capacity building, trust

## Abstract

We use a community based participatory research approach to examine the processes of collaboration and communication, as well as the relational interactions of one community focused health promotion coalition, the Community Faces of Utah (CFU). We assess the evolution, structure, successes, and challenges of the coalition, comprised of five distinct cultural communities, a state health department, and a university. Researchers from the university collaborated with the coalition to find that CFU is an equitable, collaborative partnership of diverse leaders that functions successfully. Shared values and trusting relationships emerged over time, forming the basis for group interaction. A community liaison to facilitate interaction and collaboration was an essential element of the success of this partnership. The experience of CFU can guide other multi-sectoral partnerships in developing functionality consistent with achieving community driven objectives.

Previous research has described the key factors for coalition formation and functioning to effect change. However, investigators have called for systematic study of the interactions and dynamics of coalition members in long-term, real-world relationships (Behringer, Southerland, & Plummer, 2018). Gaps remain in understanding how organizational climate is established and maintained, including conflict-resolution and decision-making processes, member-member and member-staff relationships, and patterns and processes of communication (Butterfoss, Goodman, & Wandersman, 1993).

Here, we describe a study that builds on the coalition literature by investigating the relational dynamics and communication of one community-focused health promotion coalition. The Community Faces of Utah (CFU) brings together organizations representing five culturally diverse ethnic communities, an academic medical center at a university, and a state health department. Over time, CFU members became aware that they had established a functioning multi-sectoral coalition in which members with competing demands and priorities could collaborate around common interests. All partners expressed interest in sharing CFU’s approach so others could learn from their experiences, which led to the study reported here. We framed our research around the following questions: (1) What is the CFU model? (2) How was the coalition formed? (3) What were early successes, challenges and solutions experienced during the formation of this partnership? and (4) Which organizational aspects of the model have changed across time? Our study examined the establishment and ongoing functionality of the CFU partnership, with a focus on relationship building, to serve as a model for guiding other multi-sectoral partnerships.

## Coalition Background

Community coalitions—defined as alliances of different organizations, such as community groups, universities, businesses, and local government officials working cooperatively and synergistically—are widely used and effective vehicles for improving local community health (Butterfoss et al., 1993; Kegler, Steckler, McLeroy, & Malek, 1998; Zakocs & Edwards, 2006). Achieving a successful coalition requires considerable effort and diligence from all partners. The literature on key elements of successful coalition formation and functioning is substantial.

The process of coalition formation impacts eventual success (Butterfoss, Lachance, & Orians, 2006). Coalitions often form in response to a funding proposal or from existing coalitions, and formation is typically initiated when a “lead agency with links to the community brings together key organizations that recruit a group of community partners” to work on a health or social concern (Butterfoss et al., 2006, p. 23). Initial strong, committed, and visionary leadership with the capacity to mobilize members and the skills to facilitate shaping a coalition’s mission and goals are key for eventual functionality (Butterfoss et al., 2006). Also vital are early administrative and management infrastructure, core membership with previous experience in coalitions and/or with the health issue(s), and a clear coalition mission (Butterfoss et al., 1993; Butterfoss et al., 2006). Florin, Mitchell, Stevenson, & Klein (2000) found that coalitions that focus on building their own capacity during formation, such as creating a task-oriented social climate, increasing members’ skills, and creating links with member organizations, were more likely to produce effective community health interventions than programs that did not build capacity at the beginning. Behringer et al. (2018) reported that group identification and review of expected benefits and contributions facilitated successful coalition formation.

A standard measure of coalition effectiveness is community wide change. Research is clear on the key contributing factors to effective coalitions. The findings of Butterfoss et al. (1993), Kegler et al. (1998), and Zakocs and Edwards (2006) align with those of Foster-Fishman, Berkowitz, Lounsbury, Jacobson, and Allen (2001) in identifying four “collaborative capacities” that lead to effective coalition functioning and sustainable community change. These are: (a) *member capacity* (e.g., positive intergroup understanding, diverse membership, member training, knowledge sharing, members with needed skills, and diversity of member competencies); (b) *relational capacity* (e.g., positive intergroup interactions, group norms, superordinate shared goals, inclusive decision-making processes, and external relationships); (c) *organizational capacity* (e.g., task focus, formal roles/processes, infrastructure, skilled staff, strong leadership, and an outcome orientation); and (d) *programmatic capacity* (e.g., community input and innovative programs).

### History of the Coalition: Community Faces of Utah

CFU is a long-term partnership among five diverse culturally based community organizations (six members); the Utah Department of Health (UDOH, two members); and the University of Utah School of Medicine (UUSOM, three members). The participating community partners are the Best of Africa (African), Calvary Baptist Church (African American), the Hispanic Health Care Task Force (Hispanic/Latino), the National Tongan American Society (Pacific Islander), and the Urban Indian Center of Salt Lake (American Indian/Alaskan Native). One or two community leaders represent each of the five community organizations in CFU. The members representing UDOH were, at the time, staff for the former Utah Diabetes Prevention and Control Program. In 2013, this program was combined with the Heart Disease and Stroke Prevention Program, the Physical Activity, Nutrition and Obesity Program, and School Health to form what is now the Healthy Living through Environment, Policy and Improved Clinical Care (EPICC) Program. The UUSOM members include the faculty co-directors and community liaison of the Community Outreach and Collaboration Core of the Utah Center for Clinical and Translational Science.

CFU was established in 2009 when the UUSOM received funding for a one-year Community Genetics Forum project that supported education in diverse communities about the link between genetics and health. The Forum project built on a previous five-year Consumer Genetics Education Network (CGEN) project (Stark, Giles, & Johnson, 2008). The UUSOM and UDOH partners in the CGEN project first recruited a Hispanic/Latino Community Advisory Board, which included the president of the Hispanic Health Care Task Force (HHCTF), who was a breast cancer survivor with an interest in genetics and genetics education. The partners adapted curriculum materials to be culturally appropriate for the community on heredity for fifth grade students (Powell, Drits-Esser, Malone, & Stark, 2018a; Powell, Malone, Drits-Esser, & Stark, 2018b) and family health history for high school students (Genetic Science Learning Center, 2015). The group also developed new materials for students to take home for family education. UDOH next approached the National Tongan American Society (NTAS), with whom it had partnered on other health education projects, inviting them to participate in the CGEN project. NTAS and its Community Advisory Board collaborated in selecting and adapting materials from the heredity and family health history materials to be culturally appropriate for use in its adult health education classes.

When the funding opportunity for the Forum project was announced, the UUSOM principle investigator met with the HHCTF and NTAS leaders to discuss whether they would be interested in collaborating on such a project, and if so, how to structure it. They expressed a strong interest in the project and recommended that it involve several community organizations, each of whom would receive a $5,000 mini grant to hold community education events, culminating in a Forum with all of the communities (a Forum event was required for the funding). After the project was funded, five organizations in addition to HHCTF and NTAS were invited to participate. During 2009–2010, each community group held one or more “mini forums” for its members, focusing on family health history as a way to identify hereditary health issues in families, and providing education about common chronic diseases that have an inherited (genetic) component and are of highest concern in their community. The leader from the Urban Indian Center coined the term “inherited health,” since the word “genetics” had negative connotations in his community, which the other communities adopted.

The community organization leaders, UDOH and UUSOM partners met each month to share what each organization was planning for its mini forum(s), share lessons learned from these events, and to collaboratively plan the culminating, all-day Genetics and Health Forum (Davis et al., 2010). The community leaders decided that the Forum should focus on cancer, diabetes, and heart disease, which they identified as health issues facing each community. To honor the storytelling that is part of their cultures, the session on each disease began with a community member describing his or her experience with it. A physician then described treatments and a researcher discussed current research efforts in that field. A separate children’s program focused on activities for healthy living, and allowed parents to attend the Forum. At the end of the project, five of the seven community organizations decided that they wanted to continue working together and meet monthly, despite a lack of continued funding. Of the two organizations that decided not to continue, one was a small group that operated across several states, and the other was a church group that had not previously focused on health.

An important part of the process for solidifying CFU was the collaborative development of the group’s mission statement, written during its first retreat, several months after the coalition was initiated: *Community Faces of Utah (CFU) is a nexus of communication between resource sources and those who need a voice to achieve better health*. *CFU is dedicated to creating partnerships and engaging in opportunities for entire communities to become healthier*. *We work together to better provide health promotion and education for all people who face health disparities*. During the retreat, CFU established the following goals: (a) build capacity within communities; (b) tailor communication within communities/partnerships; (c) provide health education for communities/partnerships; (d) provide health access by linking communities and partners to resources; (e) provide interpretation and translation services for communities/partners; (f) impact health policy that creates a healthier environment for communities; (g) reduce health disparities; and (h) become a self-sustaining organization. The mission statement and goals were initiated with a brainstorming session in which everyone’s ideas were recorded on a large white board. These ideas were then grouped and refined through a facilitated discussion.

In 2011, approximately seven months after the Forum project ended, CFU was approached by the UUSOM Center of Excellence in Women’s Health who proposed a partnership with CFU in applying for a six-year Office of Women’s Health grant to address obesity among women from communities facing health disparities (one-year planning and five-year implementation). The CFU community leaders identified obesity as a health challenge in all of their communities and agreed that the proposed project was consistent with the CFU mission. The investigators used an inclusive approach throughout the Coalition for a Healthier Community for Utah Women and Girls (UWAG) project by engaging CFU in collecting preliminary information from each community, planning and conducting the project, analyzing the data, and disseminating the findings (Simonsen et al., 2015). Several women from each CFU community were trained as “community wellness coaches” (a form of community health worker) and collectively recruited over 400 women to participate in 12 months of lifestyle coaching. The participants made statistically significant improvements in their own physical activity behaviors, fruit/vegetable consumption, sleep, and depression, in addition to reporting positive improvements in family members’ health behaviors (Simonsen, 2016). In addition, the CFU community organizations implemented new guidelines supporting healthy food and beverages at events. This project provided continued momentum and focus for the coalition and led to additional collaborations with researchers, including several related to HPV vaccination (Kepka et al., 2018; Lai et al., 2017).

## Methods

We initiated a study to examine the formation, functionality, challenges, and successes of CFU in 2012. A UUSOM researcher and a graduate student facilitated the research. The lead researcher holds a Ph.D. in educational psychology, with training in qualitative, quantitative, and mixed methods research, and had served as an external evaluator for some of CFU’s earlier projects. The graduate student was in the UUSOM genetic counselling master’s degree program and had a graduate assistantship at UUSOM. The researchers became close to the CFU members during the course of the study and eventually felt accepted as an extended part of the CFU “family.”

### Participants

All 11 CFU members from the three types of partner entities—community groups (n = 6), UUSOM (n = 3), and UDOH (n = 2)—were study participants. Members were classified as those who attend the CFU meetings regularly and who were responsible for making decisions on behalf of their communities. Two of the UUSOM members include the UUSOM faculty member and community liaison who initiated the coalition.

### Research Design

We used the framework of community based participatory research (CBPR) to conduct this research, from identifying research questions to publication (Viswanathan et al., 2004). CBPR complements more traditional research approaches, in which university researchers develop and conduct all aspects of a research study (Nyden, 2003). CBPR equitably links academic and community partners, thereby making it an appropriate approach for studying partnerships between these entities (Minkler, Blackwell, Thompson, & Tamir, 2003). In CBPR, community members bring keen expertise—such as knowledge of community views and cultural insights—that is often unknown to those outside of the community (Christopher, Watts, McCormic, & Young, 2008). At a monthly CFU meeting, the lead researcher presented a draft of key study goals that was based on prior CFU discussions and initial research objectives. After considerable discussion and revisions, CFU members agreed on research objectives and methods. At subsequent monthly CFU meetings, the lead researcher provided status updates with time for discussion throughout the data collection and analysis process.

### Data Collection and Procedures

We developed the research instruments collaboratively through group discussion. The researchers presented suggestions for appropriate data collection measures and received input from CFU. We agreed upon three measures, which the researchers developed: (a) individual interviews; (b) focus groups; and (c) a survey that was completed anonymously. We used the three data sources for triangulation; each measure elicited information from a different angle or perspective. The qualitative data (interviews and focus groups) provided the richness and depth of responses, while the survey provided anonymously collected data that helped to decrease response bias.

To gather evidence for content validity (Bass, Drits-Esser, & Stark, 2016; Reeves & Marbach-Ad, 2016), two UUSOM CFU members and an outside UUSOM researcher who was working with CFU on a separate project, vetted each instrument. They determined that the intended constructs for measurement were appropriate for addressing the study research questions, and that the survey items and interview protocols were relevant to those constructs (Gehlbach & Brinkworth, 2011). In addition, for the survey, the researchers established response process evidence for validity through conducting cognitive interviews, in which two members of the communities represented by CFU, thought aloud as they addressed each survey item (Bass et al., 2016; Reeves & Marbach-Ad, 2016). Items were refined based on this feedback.

#### Measures.

The eight-item, semi-structured individual interview protocol allowed the researchers to gain an in-depth understanding of individuals’ perspectives of the CFU processes (See Appendix A). The 1 to 1.5 hour interviews included questions about how leaders’ roles have impacted their community’s health awareness, feelings of equality within CFU, and key elements of building the partnership. The lead researcher conducted the individual interviews over a five-month period. We designed the six-item, semi-structured focus group protocol to obtain an understanding of the group dynamics as well as the synergy between each individual and the partnership. Questions included motivation for continuing to work together as a group, group challenges, and reasons for participation (See Appendix B). The researcher held a 1 to 1.5 hour focus group with each of the three types of CFU partner entity (i.e., Community, UUSOM, and UDOH). The focus groups and interviews were audiotaped and transcribed.

The lead researcher administered a 39-item CFU Partnership survey to the CFU members via pen-and-paper during a monthly CFU meeting. The survey used a 5-point Likert-type scale (5-strongly agree, 1-strongly disagree), which asked participants the extent to which they agree with each statement *currently* and how they would have responded *at the time they first joined CFU* (See Appendix C). The survey served a dual purpose: (a) to provide corroborative data to confirm or disconfirm themes identified in the qualitative analysis; and (b) to provide quantitative data that measured changes across time. Surveys used in other coalition and CBPR-oriented studies (Bell-Elkins, 2002; El Ansari, 1999; Lehman, 1999) informed the survey’s development. It was divided into 4 subscales: (1) *organizational practices*; (2) *member participation*; (3) *participating organizations and diverse communities*; and (4) *technical support*.

Approval from the UU Institutional Review Board was received in March 2012 (IRB_00055016). Consent documents were reviewed with participants, and consent was obtained prior to formal participation.

### Data Analysis, Data Validation, Authorship Requirements

We used qualitative techniques to analyze the interview and focus group data. First, the researchers identified broad categories in the data by reviewing interview and focus group transcripts, and as they reread the transcripts they refined the categories (Miles & Huberman, 1994). For each category, we engaged in a cyclical process of analyzing and writing about the data. After developing memos on each category, the researchers identified and grouped themes around the research objectives. CFU members individually reviewed and provided written comments on the themes. For survey data analysis, we used SPSS Version 25 to produce descriptive statistics, and to conduct repeated-measures *t* tests and Wilcoxon signed-rank tests to assess changes across time. When applicable, the survey results confirmed themes already developed in the qualitative analysis, and we resolved disconfirming evidence through clarifying discussions with CFU members. The researchers refined themes based on this feedback, CFU reviewed them a monthly CFU meeting, and additional revisions followed.

The CBPR approach inherently fosters data validation because of the significant community member involvement in the research process. We used data triangulation, including three methods of data collection, and a mixed-methods design to further support the validity and credibility claims in this study (Guba & Lincoln, 1989).

While the CBPR literature is robust, a minority of these publications describe coalitions where community partners contribute to each phase of the research process, especially in issues of authorship. The group collaboratively decided on authorship guidelines, informed by community engagement professional journal guidelines, which the lead researcher presented to the members at a monthly CFU meeting. Authorship was defined as contributing to study design, discussion of qualitative themes, and revisions to the written manuscript. All 11 partners met the criteria for authorship.

## Results

The following sections describe the themes that emerged from the data and provide representative quotations. Themes associated with the CFU approach, including structure, function, and evolution, are described first, followed by those associated with CFU successes and challenges. The survey results follow.

### The CFU Model

#### True collaboration and commitment to equity.

Members reported that CFU is a true collective with shared goals, concerns, values, and a group identity.

The key for CFU success is considering each other and respecting each other. We believe that each side, either University, Communities or Health Department, has something to contribute. Each side brings their strength, opinions, information, and we put it together, instead of going just with one side (Valentine, Community).

The partnership shares a deep commitment to equity, defined by the group as valued the same but allowing for different contributions among all members. Each member described feeling valued by the group because of his or her unique contributions. As Louisa (UUSOM) explained:

The Community members are bringing…their expertise in their communities. The University side is bringing resources, money…the research skills that help us get the bigger grant money…. The Health Department brings its expertise in engaging the public in improving health.

Members explained that the differences in their skills and backgrounds have been essential to the success of CFU. For example, “We all bring a different set of qualifications, a different kind of experience and information, a different kind of training, but everybody’s listening to everybody, and we find that all of that has value in making good decisions” (France, Community).

#### Equality as group norm.

The focus on equality is evident in the structure and organization of the monthly meetings. “We don’t have a formal structure—we don’t have a chair, vice chair, secretary. [The community liaison] creates the agenda and our meetings basically run themselves” (Ed, Community). Decisions are made by consensus, following the standard of group congruence rather than majority based voting. Final decisions usually involve a blending of ideas:

When we have something to discuss, we put it on the table. We discuss and make sure everybody understands and agrees. We treat each other with respect and I feel like we have equality, like when there is something to discuss, we don’t vote. We keep on discussing it and finally come to an agreement (Valentine, Community).

In other words, decisions are made by congruence, not compromise. Participants reported feeling respected by others in the group and having their voices heard. “My opinion matters in whatever decision we take” (Sylvia, Community). Dorienna, a community member, added, “Everyone has their own opinion, everything is weighed.”

### Development of the CFU Partnership

#### Recruitment and member characteristics.

CFU evolved from a group of organizations brought together to carry out a single project, as previously described. One UUSOM faculty member had previous links to the community organizations and was respected as an advocate for community health promotion. Sylvia, a community member, explained, “It was important to trust the person that’s recruiting [from the UUSOM]… It was my [already established] relationship with Louisa that did it and a total trust in what she was doing.” After the project was funded, Louisa hired a community liaison, Heather, who had previous experience as an elementary teacher in a Title I school serving students with diverse demographics, as a school administrator, and as a clinical instructor in the UU College of Education. Heather brought strong interpersonal communication skills to her work, including her ability to facilitate conversations and negotiations with multiple types of individuals (e.g., children, parents, teachers and school administrators) and build safe classroom communities with students from many cultures (Salt Lake City is a refugee resettlement center). She also was aware of social determinants and how they can impact a child’s health and ability to learn. While she is employed by UUSOM, her primary orientation is that of a community organizer; the CFU community members trust her to advocate for them and serve as their voice when they are not present.

When CFU was in the process of formation, the community liaison recruited additional community leaders to participate. These leaders were identified through contacts made in her prior work and via referrals by a UDOH staff member. She met with each community leader, described the project, and invited them to participate. Members were already well-known and respected leaders in their communities. They are “the community gatekeeper… people that are so well connected to the community that they could take a message back to the community” (Brenda, UDOH). Another member explained the necessity of having established leaders: “If you’re not known in your community, it’s going to be very hard to get something like CFU started…it would be like pulling teeth to get somebody to come along” (Doriena, Community). Established leaders know their communities well and know what will and will not work for them.

CFU leaders share certain key qualities, including a willingness to risk doing something new and different. Ed, a community member, explained, “The leaders are self-actuated—they don’t wait around to be told what to do.” They are humble and are willing to listen and learn from one another. Community member France described the members as “open-minded, humble people who really want to work together, collaborate, share resources, and not come with an agenda.” Leaders have passion and commitment toward improving the lives of members of their community and see the CFU partnership as one way of accomplishing that. “Everyone is in the organization not for their own good or aggrandizement. They’re here because they’re committed to what they do” (Ed, Community).

#### Relationship building.

The CFU partnership was built on a strong foundation of trust. Trust was established and reinforced through continuous positive experiences with members following through on promises and being accountable for actions, having goodwill toward one another, working to contribute to the betterment of other members’ communities, and having one another’s best interest at heart. Valentine, a community member, explained:

We always do the things in the way we agreed on doing it. That’s how I build my trust…. Information can come from [the UUSOM and UDOH] and we are able to come to an agreement because we trust each other. Because of that trust we can agree.

Another member explained:

We like each other. We get along. We know each other. We’re interested in each other. We have the other members’ best interest at heart. They have ours. That’s something that I think can’t be overstated, in terms of the importance of building those relationships (Steve, UUSOM).

Further, trust grew because the members understood that growth and success takes time and that it is not always a smooth process.

When asked why they made the decision to join, Community members reported recognizing that the UUSOM faculty member and community liaison intended to form an equitable partnership. The community members explained:

We jumped on this because they came with an open mind already. They wanted to work with us to develop this partnership where everybody is equal…. [None of us] wanted to be used again…by groups that just wanted to be associated with the Community and then we never heard from them again (Community, Focus Group).

Additional reasons for joining included positive previous experiences with the UUSOM leaders, a current relationship with some of the other community leaders, and one member indicated an interest in genetics.

#### Skilled facilitation.

The findings unambiguously revealed the importance of an effective community liaison to facilitate the partnership’s work. For CFU, the liaison’s primary role is to ensure that relationships among the partners are supported. Her tasks include communicating with members about CFU’s work; organizing meeting agendas; scheduling additional meetings; facilitating CFU members’ work on projects; and meeting with current and potential external researchers and collaborators to discuss projects along with educating them about CBPR and working with communities; and helping resolve any issues that arise. She communicates constantly with CFU members via email, phone, text messages, and in-person meetings. She also attends events in each community:

A community liaison is critical because nobody else in the group has that time. She meets with people individually. Before every meeting she’s already talked to everyone about the agenda and prepared them for the meeting so there aren’t any surprises… People are prepared to have a thoughtful discussion. We increasingly have people who want to talk to CFU so Heather meets with them and educates them about working in a community engaged way (Louisa, UUSOM).

Fahina, a community member, explained, “She is the point of contact in all things—it’s quick and easy.” Heather (UUSOM) described her activities as:

You have to check in on people: ‘How’s your day going? Are you still able to attend?’ I think that’s an important lesson for people to realize when working with the different communities. It requires more intense communication and a lot of different modes [of communication]…. Also, it’s key to have that agenda to get through the two hours we have monthly.

#### Other contributing factors.

Having a starting project (the genetics and health education project) provided a framework to develop the CFU group identity and process. “We needed a strong goal…that solidified CFU and gave us credibility and a common identity” (UDOH, Focus Group). The goals identified by the group typically aligned with the goals of the separate community member organizations. As CFU evolved, it was able to undertake several projects, including the large-scale project on obesity reduction that further defined and refined the partnership, its mission, and its work as a united entity.

Other factors that contributed to the growth, cohesiveness, and productivity of the partnership included requiring members’ regular attendance at all meetings, and having a consistent and welcoming place in which to hold meetings. It also has been important to hold CFU-sponsored events, such as health fairs, at locations that are convenient for the members of the communities the leaders represent. Next, we report on the successes and challenges the group has experienced.

### CFU Successes

#### Each community benefits.

Each partner reported that his or her community benefits from involvement in the partnership. It is understood that knowledge and available resources are shared. By being exposed to the diversity of people and organizations represented in CFU, all members feel they are broadening their resource base. The community members reported increased health awareness and knowledge of available resources, among other benefits for their respective communities. “There are resources and all kinds of things that we, as community, tap into” (Fahina, Community). Further, members from the communities are experiencing a growth in trust and comfort with members of other communities. As Doriena, a community member, explained, “Our communities are becoming more trustworthy with each other as well as the [leaders] trusting one another.” Ed (Community) explained, “It’s been an opportunity for me to take the stories of [members of my group] and their situations out of their circles.”

The UUSOM members and some of their colleagues have gained opportunities to understand how UUSOM resources could be leveraged to serve communities. Louisa (UUSOM) explained, “This certainly raised awareness within the CFU UUSOM leadership as well as the colleagues we interact with here at the university, about health disparities.” The university members have been better able to make meaningful connections between academia and communities. Fahina, a community member, observed that this “is a rare opportunity where researchers on the university level have the community readily available and can implement research and evidence-based programs directly.”

UDOH members and some of their colleagues have gained an understanding of effective strategies and methods for attaining their public health goals in underrepresented communities. Further, they have learned to alter their goals in response to the reality of the needs and cultural specifics of the communities. Brenda, UDOH, explained:

I think just listening to some of the challenges and barriers that people in CFU face has really been an eye opener…. It’s been very helpful for us to step back and rethink and look at our interventions again and let the community have more say and more voice in what we try to implement to help people become healthier.

Further, members have shared this knowledge with receptive UDOH colleagues.

#### Professional and personal growth.

Members felt that they gained important and meaningful lessons in leadership and intercultural skills through their participation in CFU. For example, “Working with other communities and seeing how they handle things has helped me to better myself and how I handle different programs or obstacles” (Doriena, Community). Some reported an improvement in their leadership skills. One community member explained:

I became a better leader because I’m able to provide more resources and more information to my community. I feel stronger because I have people who are supporting me, people I work with, people who can advise me. I feel better that I have support from CFU (Valentine, Community).

The UUSOM members felt they increased their understanding of the health needs, challenges, and other realities of the communities represented by CFU. This gained knowledge has enabled them to more genuinely and effectively serve the needs of these communities. One UUSOM member explained:

The benefit we get out of this is that while we understand the academic and scientific world and the theoretical world of health, what we and other universities have failed to understand is the day-today reality of health for the people that we’re supposed to be serving…. So it’s coming together with these communities and hearing that voice and that feedback that allows us to truly face…what the reality is and have our partners help us understand what it is that we need to be doing to serve the needs of our society (Steve, UUSOM).

CFU members reported they have become more open toward and tolerant of others who are members of different ethnic communities. “My view used to be that this is a Black issue, or this is a Hispanic issue, but no longer is that the case” (Community, Focus Group). Some have experienced an increased comfort level with other groups, including ethnic communities, UUSOM people, and UDOH people. For example, France, a community member, explained:

It’s given me more of a sense of inclusion, of the need to work with people who have, at least on the surface, differences. And it’s also helped me to understand better that we have more in common as human beings than we have that’s different. And so I think I’m striving to be a better leader in that sense, and a leader that does not make decisions based on a particular group or community organization, but rather to be more inclusive of other groups.

Additional feedback from other CFU members suggested that individuals have become more humble through engaging in the CFU interactions. Others have gained new personal strength. Several members have been inspired to further their education.

The CFU members consider one another good friends, or even family. Ed, a community member explained:

When I think of what describes my feelings for our group, a Maori word comes to mind, ‘Aroha.’ It’s one of those words that cannot be adequately translated into English…it means love in the fullest context of the word. It means respect, honor, caring, comforting, connecting, embracing…and more.

#### Empowerment and group recognition.

CFU members felt that there were goals they could accomplish as a group that they could not achieve individually:

There’s actually power in numbers and so for [our individual communities] to complain about something, we get labeled as ‘there they go again,’ but when all of us come together…. it’s very powerful when five groups of us, you know, are raising the issue (Community, Focus Group).

Because of their unified goals and message, members, in different ways, felt they gained legitimacy and credibility, and that their voices were now being heard by policy makers and other decision makers. Further, the community members felt they could represent their communities in the public sphere more effectively because of the partnership. “Coming together from different points of view and perspectives, and not just one group yelling and making squeaky noises, but all five groups making a noise and together…. We squeak in harmony!” (Community, Focus Group). UUSOM and UDOH members felt they were bolstered by working closely with people in the communities. Therefore, their messages to their colleagues were representative of realistic community needs and challenges.

CFU’s reputation has grown significantly. Groups—including other community organizations, researchers, and program leaders—regularly request some level of partnership with CFU. As Fahina (Community), explained, “CFU has the option to accept or reject [requests for partnership] based on pros and cons.” Thus, the group is gaining recognition and credibility for its productivity and successes. CFU members regularly submit funding applications as a single entity, a process that leads to stronger applications and higher chances of funding. Some CFU community groups have emulated the CFU partnership model with other groups within their community. “Other people are following this model in their communities, which shows that it is spreading and really working” (Doriena, Community).

The partnership has been spreading its message. CFU has provided a “forum for our voices…. It’s opened other doors with other organizations and health sciences” (Ed, Community). The group is making powerful impressions. It is regularly invited to present and/or to participate on panels at meetings and conferences, and several members have made local, state, national and international conference presentations on the strengths and successes of CFU and its work. “We’ve gotten invitations from the [UUSOM members’] colleagues to some of their conferences that we would not have been considered for prior to CFU. They wouldn’t have known us and even if they did, they would have ignored us” (France, Community). Grant, from UDOH, described how its division director has attended CFU presentations: “She’s listening and she’s a good one to advocate for the group.”

### Challenges to the CFU Partnership

#### Developing trust.

As with any important relationship, learning to trust took considerable time, c ommitment, and effort. It took several years for the members to develop the level of intragroup trust currently experienced. “We were feeling out to what extent we could rely on the other folks around the table. That developed over time, to the point that we can now deal with some pretty tough stuff” (Steve, UUSOM). One community member described his initial uncertainty about working with UUSOM leaders: “One of the reluctances that we had at the beginning was that the people at the university were going to do a show up, ask questions, collect information, and disappear. And it took us time to establish the relationship with them” (France, Community).

#### Learning to work together.

All members invested their time to learn to make decisions that were mutually agreed upon. Over time, the group has become more efficient. “We brainstormed a lot at the beginning of CFU…to find out what we all had in common with health issues” (Doriena, Community):

In a meeting now, it would take five minutes to make a decision. It probably took us two months to get through that process at the beginning. We came out the other side recognizing that here are some new ways we could do things to help avoid that in the future (Steve, UUSOM).

The group experienced growing pains as the partnership developed. As may be expected, bringing so many different perspectives together opens the door for disagreements. The community liaison described her role during the evolution of CFU as averting potential conflicts:

Some of the first efforts took time and we had people get upset, we had side meetings, we had to work through a lot of things, we had to work with individuals…. Now it’s to the point where if someone has an issue they’ll just say it and we have this sort of trust built up…a shared knowledge base that we all draw from.

The community liaison addressed issues and challenges as they arose by contacting members, often individually. “When there was any type of feeling of conflict or tension, [the community liaison] would immediately speak to one of the members…communication, clarification, ask the questions again and again and it seems to allow that conflict to not happen” (UUSOM, Focus Group). Potential conflicts, therefore, were readily addressed by quick communication and clarification. From the beginning, the community liaison set the tone and reinforced a group norm of equality and equity. “She keeps her finger on the pulse of everything, she ensures that everybody feels equal…. She set this tone saying everybody has the same voice, everybody feel free, we’re not going to judge and we’re going to respect everybody” (UDOH, Focus Group).

#### Accountability to own community.

Some leaders felt that they needed to prove to their own organizations that their time commitment and effort in CFU was justified. Sylvia, a community member explained, “When we first started and I introduced it to my organization I had people say, ‘Why would you want to do work to better the lives of other people when our community is not doing well?’” However, as CFU began experiencing successes and the benefits of this partnership spilled over into individual communities, fewer members questioned the leaders’ participation.

#### Resources.

CFU members consistently reported that time and funding are in chronic short supply. All of the members hold positions outside of CFU, and therefore the time required is difficult to add to their already busy schedules. “I have heard from everybody that they’re also facing the challenge with time” (Valentine, Community). Securing adequate funding to conduct the work CFU would like to accomplish is a persistent challenge.

### CFU Partnership Survey Results

The survey results for each category for present-day CFU membership (how members felt at the time of the study) supported key findings from the qualitative data. Response averages for the *organizational practices* category indicated that participants either agree or strongly agree with all items (range: 4.0–5.0) (e.g., the vision, mission, and goals are CFU are visited regularly; CFU conducts meeting in an organized manner; partners regularly participate in CFU meetings; the vision, mission, and goals are obvious to others outside of CFU). The results for *member participation* (e.g., all partners have input into CFU decisions; bi-directional learning occurs within the partnership; pride in CFU accomplishments) indicated that participants’ responses averaged toward strongly agree (range: 4.6–5.0). When asked if CFU prioritizes the needs of any one partner, response averages were between strongly disagree and neutral (range: 1.5–2.7). For the category of *participating organizations and diverse communities*, participants agreed or strongly agreed that CFU impacts the health practices of members’ communities; CFU helps raise public awareness of health issues; CFU develops collaborative relationships with external organizations; partners are focused on creating positive health changes for their community/organization (range: 4.1–5.0). Finally, in the *technical support* category, results revealed that participants were neutral on whether they had sufficient funding and enough space to conduct their community activities (range: 3.1–3.5). They agreed there was enough space to conduct CFU meetings and that members had sufficient training (range: 4.4–4.5).

Participants’ retrospective survey results, which compared responses from when they first joined CFU to to the time when the study was conducted three years later, revealed statistically significant increases over time at an alpha level of .01 for each subscale (See Table 1).

One exception was the CFU member participation category, which showed a small, non-significant increase over time. CFU members, then, perceived coalition growth and change in three key areas (organizational practices, participating organizations and diverse communities, and technical support) from its inception to three years later as a functioning, successful coalition. The members perceived little change in the category that asked about the presence of bi-directional learning, all partners having input in CFU decision making, prioritization of one group over another, and consistent patterns of communication and decision-making. Implications for these findings are described in the following section.

## Discussion

In this study, we employed a CBPR research framework to: (a) identify and describe the formation and functionality of one successful community coalition that can be used as a model for developing similar coalitions, (b) examine and describe the interpersonal dynamics of group membership to address gaps in the literature, and (c) advance the science of developing health-focused coalitions by investigating the successes and challenges of CFU. The results suggest that CFU is an equitable, cohesive, and collaborative partnership of diverse leaders and organizations that functions productively to achieve group goals. CFU reports high member satisfaction, shown to be linked to leadership skill, staff skill, communication, and task focus (Kegler et al., 1998).

CFU members have experienced important, meaningful successes that include increased awareness of available resources, professional and personal growth and empowerment, group recognition, a local and national presence, and close friendships. The group’s successes impact all five health disparity communities, most of which are large enough to contain sub-communities that also benefit from CFU’s successes. Further, this partnership has allowed diverse communities to be heard as a unified voice. This is particularly important in a state with a population that is predominantly non-Hispanic White (78.5%) with smaller percentages of other races/ethnicities – 14.0% Hispanic or Latino, 1.1% Black or African American (non-Hispanic), 1.0% American Indian or Alaska Native (non-Hispanic), and 1.0% Pacific Islander (non-Hispanic) (percentages do not include individuals who reported belonging to more than one race), (Utah Department of Health, 2015). The study findings suggest that members’ perceived benefits outweigh the costs (Butterfield et al., 1993). Each partner reported benefitting from experiences and lessons learned from other CFU partners, creating opportunities for multi-directional learning, and having a substantially wider net of wisdom. In addition, each partner group benefits from the variety of political and personal connections that members share with one another (Behringer et al., 2018).

The partnership faced challenges, or growing pains, as it developed into today’s productive and cohesive group. The challenges included developing trusting relationships, resolving potential conflicts through communication, learning to work together and agree on common goals, navigating constraints and allocation of limited time and funding, and maintaining accountability to the communities the members represent. These challenges at times threatened to impede achievement of the group’s goals. Most have been resolved; nevertheless, members continue to face the challenges of insufficient resources.

The findings show that CFU’s formation and functionality meets Foster-Fishman et al.’s (2001) four key capacities of effective partnerships that lead to community change: *organization*, *programmatic*, *membership*, and *relational* capacity. In the organizational and programmatic categories, CFU focused on building capacity during formation through initial connected, committed, visionary leadership that allowed the group flexibility to define itself over time (Butterfoss et al., 2006). The group developed infrastructure; formalized a clear mission statement, goals, and roles for community health engagement; and had a consistent task-oriented working climate (Butterfoss et al., 1993; Butterfoss et al., 2006; Florin et al., 2000). Other logistical factors for CFU’s capacity building included beginning with a project upon which to build group identity, holding meetings at a consistent time and place, requiring regular attendance at meetings, having a welcoming and inclusive meeting space with easy access for all members, and ensuring that the community liaison was available to coordinate activities. Alignment to the other two key capacities posed by Foster-Fishman et al. (2001) is described next.

### Relationship Building

Consistent with other research, the study results showed that the building of trusting relationships among members over several years while working together on goal-driven projects was central to coalition functionality and effectiveness (e.g., Zakocs & Edwards, 2006). Factors that influenced the building of CFU’s membership and relationship capacities (Foster-Fishman et al., 2001) over time included inviting members who were diverse, qualified, respected in their communities, deeply committed to achieving and maintaining group equity, and committed to the coalition outcome goals. Invited members brought different, appropriate resources and skills, and CFU provided opportunities for training (Butterfoss et al., 1993). Further, the collaboration and communication patterns the coalition used led to positive perceptions of intragroup relationships, feelings of cohesion, fruitful decision making, and smooth conflict resolution (Butterfoss et al., 1993).

The community liaison prioritized relationship building as she supported the community members in carrying out their project responsibilities, facilitated communications between the community members and researchers, organized meeting agendas, prepared each community leader for meetings, and facilitated meetings in ways that ensured that everyone’s voice was heard and respected. At the onset of the coalition, at each meeting and each interaction, the liaison prioritized the establishment of member equality and ensuring that each member’s voice was heard. The willingness of members to set group communication norms, set group norms for conflict resolution, and openly address issues and challenges was a key factor in building strong, trusting relationships.

Interestingly, the retrospective survey results indicated that the constructs in the membership category (i.e., the presence of bi-directional learning, all partners having input into CFU decisions, having a voice, having consistent patterns of communication, and comfort in asking for clarification when something is unclear) were already a strong presence in the first few months of membership and did not change significantly over time. These findings, along with the qualitative findings, suggest that the presence of these relational elements is key at the inception of a coalition and acts as the “glue” that binds a coalition together in the early years as it works through challenges.

Developing the “glue” of CFU’s relationships was a process that required effort, diligence, and a focus on relationship building from all members. Factors that contributed to this included maintaining a willingness to work through initial challenges, having accountability for commitments, maintaining goodwill toward one another, keeping others’ and the group’s best interests at heart, employing patience to allow successes to begin incrementally and build in momentum, practicing humility, preserving open-mindedness, and sustaining a willingness to learn from each other.

Many of these research outcomes align with the recent work of a multi-sectoral coalition in one city that worked to eliminate disease disparities in Asian populations (Arista, Vue, Byan, Choi, & Chin, 2017). Similar to CFU, the coalition members learned to engage in discussions around navigating through relationships with other members and teaching about cultural norms. They learned to encourage multi-directional learning and the leaders widened the meeting agenda to reflect the priorities of all members. The researchers concluded that project staff and capacity-building assistance from other coalition sectors supported the coalition’s structure and relationship building, which impacted implementation of project goals.

## Conclusions

This study provides qualitative, personal descriptions of members’ experiences during coalition formation and over several years of participation. The findings describe the process of relationship building and the collaboration process of one multi-sectoral coalition that could be adopted as guiding principles for other groups interested in developing a similar partnership. Guiding principles include the following:

- Initially, seek out members who share similar values, such as equality (valuing each member the same).
- Include members who are well known and respected leaders in their community or organization.
- Establish, practice, and commit to equity (being valued the same but allowing for different contributions) and congruity among partners.
- Expect member dedication, time, effort, and commitment to the group.
- Understand that trusting relationships are built over many years of working together.
- Commit to working toward the health goals of *all* participating communities.
- Ensure support for, and access to, a capable community liaison who can facilitate communication and collaboration among all partners and who is skilled in carefully and respectfully working with coalition members to resolve any conflicts.

In sum, this research focuses on the processes of forming and maintaining a productive, cohesive p artnership among multiple, diverse communities and extra-community collaborators. CFU serves as an example of a Community-Academic Medical Center-Health Department coalition that has achieved group goals and whose members and communities experienced growth and increased capacity through their affiliation with the group. This coalition provides a model for others seeking to build such a partnership.

## Supplementary Material

Appendix A **Appendix A.** CFU Study: Semi-structured Interview Guide for Individual Interviews. Link: https://s3-eu-west-1.amazonaws.com/ubiquity-partner-network/up/journal/coll/coll-2-1-29-s1.doc.

Appendix B **Appendix B.** CFU Study: Semi-structured Interview Guide for Focus Groups. Link: https://s3-eu-west-1.amazonaws.com/ubiquity-partner-network/up/journal/coll/coll-2-1-29-s2.doc.

Appendix C **Appendix C.** Community Faces of Utah Partnership Survey. Link: https://s3-eu-west-1.amazonaws.com/ubiquity-partner-network/up/journal/coll/coll-2-1-29-s3.doc.

## Figures and Tables

**Table 1:** Pre-post *t* test results for the CFU Partnership survey.

Survey Categories	Mean (pre-post)	SD (pre-post)	*t*	Significance

Organizational Practices	41.5–7.3	11.2–5.6	4.9	0.001
Member Participation	54.2–57.3	5.9–3.0	1.6	0.13
Participating Organization, Diverse Communities	35.8–40.9	6.0–4.2	4.7	0.001
Technical Support	13.8–15.5	3.0–2.8	4.2	0.002
