# Supplementary material for: The Community Faces Model: Community, University and Health Department Partners Thriving Together for Effective Health Education
Source: Collaborations (Coral Gables). Author manuscript; Available in PMC 2021 Mar 15. (PMC7959871; doi:10.33596/coll.29)
Supplement: Appendix A — CFU Study: Semi-structured Interview Guide for Individual Interviews. Link: https://s3-eu-west-1.amazonaws.com/ubiquity-partner-network/up/journal/coll/coll-2-1-29-s1.doc. [file NIHMS1671899-supplement-Appendix_A.pdf]

Appendix A

CFU Study: Semi-structured Interview Guide for Individual Interviews

-Welcome/discuss audio recording

Research Objectives

What are the elements of the CFU Partnership that can be generalized to other [inclusive] community-university-health department partnerships?

- What is the CFU Partnership model?
- How was the CFU Partnership achieved?
- What are the successes this Partnership has experienced?
- What are the challenges this Partnership has experienced?

Interview Questions

1. How has your leadership role within CFU impacted your organization, specifically your community's health awareness?
2. How has your leadership role within CFU impacted you personally?
3. What has been your experience as a partner in the Partnership?
4. How equal do you feel the Partnership has been (groups and individuals)?
   a. What has contributed to feelings of equality or non equality?
5. What is the value/role of trust in this Partnership?
   a. Please define it for this Partnership.
6. In advising others who may want to start similar partnerships, what are the key elements that have been necessary for the success of this Partnership? Why?

7. In advising others who may want to start similar partnerships, who are key people that have been necessary for the success of this Partnership? Why?

   Prompts:

   - Need community leaders (e.g., Fahina, Pastor Davis, Sylvia, Ed, Valentine, Doriena)?
   - Need university researchers with academic background, experience and values CBPR. Can make necessary connections to academic theory in the field (e.g., Steve)?
   - Need community liaison who has time and resources to connect with all partners (e.g., Heather)?
   - Need health department partners with experience relevant to participating communities (e.g., Brenda and Grant)?

8. What else would another group need to know if they wanted to build a partnership similar to CFU?
