# Supplementary material for: The Community Faces Model: Community, University and Health Department Partners Thriving Together for Effective Health Education
Source: Collaborations (Coral Gables). Author manuscript; Available in PMC 2021 Mar 15. (PMC7959871; doi:10.33596/coll.29)
Supplement: Appendix B — CFU Study: Semi-structured Interview Guide for Focus Groups. Link: https://s3-eu-west-1.amazonaws.com/ubiquity-partner-network/up/journal/coll/coll-2-1-29-s2.doc. [file NIHMS1671899-supplement-Appendix_B.pdf]

Appendix B

CFU Study: Semi-structured Interview Guide for Focus Groups

- Welcome/discussion of recording
- Overview research and purpose of groups
- Please note: All questions should be addressed from perspective of ***health awareness*** for your communities

Research Objectives

What are the elements of the CFU Partnership that can be generalized to other [inclusive] community-university-health department partnerships?

What is the CFU Partnership model?

How was the CFU Partnership achieved?

What are the successes this Partnership has experienced?

What are the challenges this Partnership has experienced?

Interview Questions

1. What were your reasons for participating in the CFU Partnership?
2. Why is CFU effective as a Partnership?
3. What have been your challenges as a group?
4. How have you grown as a group?
5. What motivates you to continue to work together as a group?
6. How has health awareness been impacted in your community through your participation in CFU?
