# Supplementary material for: The Community Faces Model: Community, University and Health Department Partners Thriving Together for Effective Health Education
Source: Collaborations (Coral Gables). Author manuscript; Available in PMC 2021 Mar 15. (PMC7959871; doi:10.33596/coll.29)
Supplement: Appendix C — Community Faces of Utah Partnership Survey. Link: https://s3-eu-west-1.amazonaws.com/ubiquity-partner-network/up/journal/coll/coll-2-1-29-s3.doc. [file NIHMS1671899-supplement-Appendix_C.pdf]

Appendix C

Community Faces of Utah Partnership Survey

For the purposes of this research study, the Community Faces of Utah Partnership is defined as a partnership of: Community Leaders, Utah Department of Health, and University of Utah.

Throughout the survey, *Community Faces of Utah* will be abbreviated as CFU. *Partners* refers to the CFU members.

	**Thinking back to when you FIRST JOINED CFU, please answer as you would have responded AT THAT TIME.**					**Please answer to what extent you agree with each statement as CFU IS TODAY.**				
	Strongly Disagree	Disagree	Neutral	Agree	Strongly Agree	Strongly Disagree	Disagree	Neutral	Agree	Strongly Agree
**CFU Organizational Practices**										
**1. CFU has a core leadership group that organizes its efforts.**	[ ]	[ ]	[ ]	[ ]	[ ]	[ ]	[ ]	[ ]	[ ]	[ ]
**2. Partners regularly participate in CFU meetings.**	[ ]	[ ]	[ ]	[ ]	[ ]	[ ]	[ ]	[ ]	[ ]	[ ]
**3. CFU conducts meetings in an organized manner (for example, with an agenda).**	[ ]	[ ]	[ ]	[ ]	[ ]	[ ]	[ ]	[ ]	[ ]	[ ]
**4. CFU has a written mission statement that has been agreed upon by all Partners of CFU.**	[ ]	[ ]	[ ]	[ ]	[ ]	[ ]	[ ]	[ ]	[ ]	[ ]
**5. CFU's vision, mission, and goals guide decision-making.**	[ ]	[ ]	[ ]	[ ]	[ ]	[ ]	[ ]	[ ]	[ ]	[ ]
**6. The vision, mission, and goals of CFU are visited regularly.**	[ ]	[ ]	[ ]	[ ]	[ ]	[ ]	[ ]	[ ]	[ ]	[ ]
**7. The vision, mission, and goals are obvious to others outside of CFU.**	[ ]	[ ]	[ ]	[ ]	[ ]	[ ]	[ ]	[ ]	[ ]	[ ]
**8. CFU engages in strategic planning and has a long-range plan.**	[ ]	[ ]	[ ]	[ ]	[ ]	[ ]	[ ]	[ ]	[ ]	[ ]
**9. Partners identify measurable outcomes for CFU on a regular basis.**	[ ]	[ ]	[ ]	[ ]	[ ]	[ ]	[ ]	[ ]	[ ]	[ ]
**10. CFU outcomes are in writing.**	[ ]	[ ]	[ ]	[ ]	[ ]	[ ]	[ ]	[ ]	[ ]	[ ]

	Thinking back to when you FIRST JOINED CFU, please answer as you would have responded AT THAT TIME.					Please answer to what extent you agree with each statement as CFU IS TODAY.				
	Strongly Disagree	Disagree	Neutral	Agree	Strongly Agree	Strongly Disagree	Disagree	Neutral	Agree	Strongly Agree
**CFU Organizational Practices (continued)**										
**11. CFU outcomes are accessible to others outside of the Partnership.**	[ ]	[ ]	[ ]	[ ]	[ ]	[ ]	[ ]	[ ]	[ ]	[ ]
**12. CFU evaluates its own performance.**	[ ]	[ ]	[ ]	[ ]	[ ]	[ ]	[ ]	[ ]	[ ]	[ ]
**CFU Member Participation**										
**13. CFU Partners have developed consistent patterns of communication and decision-making.**	[ ]	[ ]	[ ]	[ ]	[ ]	[ ]	[ ]	[ ]	[ ]	[ ]
**14. All Partners have input into CFU decisions.**	[ ]	[ ]	[ ]	[ ]	[ ]	[ ]	[ ]	[ ]	[ ]	[ ]
**15. CFU meetings reflect an open exchange of ideas between Partners.**	[ ]	[ ]	[ ]	[ ]	[ ]	[ ]	[ ]	[ ]	[ ]	[ ]
**16. Bi-directional learning (partners learning from each other) occurs within the CFU Partnership.**	[ ]	[ ]	[ ]	[ ]	[ ]	[ ]	[ ]	[ ]	[ ]	[ ]
**17. I feel a sense of pride in what CFU accomplishes.**	[ ]	[ ]	[ ]	[ ]	[ ]	[ ]	[ ]	[ ]	[ ]	[ ]
**18. Participating in CFU takes too much of my time.**	[ ]	[ ]	[ ]	[ ]	[ ]	[ ]	[ ]	[ ]	[ ]	[ ]
**19. CFU prioritizes the needs of the University over the needs of the Department of Health and the Community Partners.**	[ ]	[ ]	[ ]	[ ]	[ ]	[ ]	[ ]	[ ]	[ ]	[ ]
**20. CFU prioritizes the needs of the Community Partners over the needs of the Department of Health and the University.**	[ ]	[ ]	[ ]	[ ]	[ ]	[ ]	[ ]	[ ]	[ ]	[ ]
**21. CFU prioritizes the needs of the Department of Health over the needs of the University and the Community Partners.**	[ ]	[ ]	[ ]	[ ]	[ ]	[ ]	[ ]	[ ]	[ ]	[ ]

	Thinking back to when you FIRST JOINED CFU, please answer as you would have responded AT THAT TIME.					Please answer to what extent you agree with each statement as CFU IS TODAY.				
	Strongly Disagree	Disagree	Neutral	Agree	Strongly Agree	Strongly Disagree	Disagree	Neutral	Agree	Strongly Agree
**CFU Member Participation**										
**22. CFU provides me with the opportunity to learn new skills.**	[ ]	[ ]	[ ]	[ ]	[ ]	[ ]	[ ]	[ ]	[ ]	[ ]
**23. I feel that I have a voice in what CFU decides.**	[ ]	[ ]	[ ]	[ ]	[ ]	[ ]	[ ]	[ ]	[ ]	[ ]
**24. I trust the other CFU Partners.**	[ ]	[ ]	[ ]	[ ]	[ ]	[ ]	[ ]	[ ]	[ ]	[ ]
**25. I ask questions and ask for clarification if things are unclear.**	[ ]	[ ]	[ ]	[ ]	[ ]	[ ]	[ ]	[ ]	[ ]	[ ]
**26. I really care about the future of CFU.**	[ ]	[ ]	[ ]	[ ]	[ ]	[ ]	[ ]	[ ]	[ ]	[ ]
**CFU, Participating Organizations, and Diverse Communities**										
**27. CFU sets priorities based on the needs of each participating community.**	[ ]	[ ]	[ ]	[ ]	[ ]	[ ]	[ ]	[ ]	[ ]	[ ]
**28. CFU designs and implements educational health experiences and programs for communities.**	[ ]	[ ]	[ ]	[ ]	[ ]	[ ]	[ ]	[ ]	[ ]	[ ]
**29. Partners are focused on creating positive health changes for their community/organization.**	[ ]	[ ]	[ ]	[ ]	[ ]	[ ]	[ ]	[ ]	[ ]	[ ]
**30. CFU develops collaborative relationships with organizations outside of CFU.**	[ ]	[ ]	[ ]	[ ]	[ ]	[ ]	[ ]	[ ]	[ ]	[ ]
**31. CFU works toward improving the quality of local health access and services.**	[ ]	[ ]	[ ]	[ ]	[ ]	[ ]	[ ]	[ ]	[ ]	[ ]
**32. CFU helps raise public awareness of health issues.**	[ ]	[ ]	[ ]	[ ]	[ ]	[ ]	[ ]	[ ]	[ ]	[ ]

	Thinking back to when you FIRST JOINED CFU, please answer as you would have responded AT THAT TIME.					Please answer to what extent you agree with each statement as CFU IS TODAY.				
	Strongly Disagree	Disagree	Neutral	Agree	Strongly Agree	Strongly Disagree	Disagree	Neutral	Agree	Strongly Agree
**CFU, Participating Organizations, and Diverse Communities (continued)**										
**33. CFU gains support from community leaders (public officials, leaders of community organizations, etc.).**	[ ]	[ ]	[ ]	[ ]	[ ]	[ ]	[ ]	[ ]	[ ]	[ ]
**34. CFU impacts the health practices of Partners' communities.**	[ ]	[ ]	[ ]	[ ]	[ ]	[ ]	[ ]	[ ]	[ ]	[ ]
**35. CFU has an impact on changing the health practices of communities outside of the Partnership.**	[ ]	[ ]	[ ]	[ ]	[ ]	[ ]	[ ]	[ ]	[ ]	[ ]
**Technical Support**										
**36. CFU Partners have the training to be effective leaders.**	[ ]	[ ]	[ ]	[ ]	[ ]	[ ]	[ ]	[ ]	[ ]	[ ]
**37. CFU has the funding it needs to be an effective organization.**	[ ]	[ ]	[ ]	[ ]	[ ]	[ ]	[ ]	[ ]	[ ]	[ ]
**38. CFU has enough space and equipment to conduct meetings.**	[ ]	[ ]	[ ]	[ ]	[ ]	[ ]	[ ]	[ ]	[ ]	[ ]
**39. CFU has enough space and equipment to conduct community activities.**	[ ]	[ ]	[ ]	[ ]	[ ]	[ ]	[ ]	[ ]	[ ]	[ ]

Thank you very much for completing this survey!
